# Editorial: Efficacy of Psychological and Psychiatric Treatments and Potential Predictors in Social Anxiety Disorder and Obsessive-Compulsive Disorder

**DOI:** 10.3389/fpsyt.2021.833131

**Published:** 2022-01-21

**Authors:** Andrea Pozza

**Affiliations:** Department of Medical Sciences, Surgery and Neurosciences, University of Siena, Siena, Italy

**Keywords:** social anxiety disorder (SAD), cognitive behavioral therapy (CBT), exposure and response prevention (ERP), obsessive-compulsive disorder (OCD), psychotherapy

Social Anxiety Disorder (SAD) and Obsessive-Compulsive Disorder (OCD) are chronic psychiatric conditions. The former is characterized by an intense fear of social situations in which the person may be scrutinized by others (1). The person fears being negatively evaluated—e.g., being judged as anxious, weak, stupid, boring, or unlikable. Such fear is generally maintained by avoidance behaviors. The latter consists of obsessions—recurrent, persistent thoughts, urges, or images experienced as irrational—and compulsions, repetitive behaviors, or mental acts which an individual feels driven to perform in response to an obsession (2, 3).

Serotonergic medications and cognitive behavioral therapy (CBT) with behavioral (i.e., exposure therapy for SAD and exposure with response prevention for OCD, respectively), and cognitive strategies (i.e., cognitive restructuring for both the disorders) have been proven to be the first-line treatments across the life span (4–6). However, amongst anxiety-related disorders, SAD and OCD are associated with the lowest remission rates, i.e., 40 and 37%, respectively (7). In addition, drop-out rates are relatively common with around 15% of patients with SAD and 9–17% of patients with OCD prematurely leaving the treatment course (8, 9).

In the light of these considerations, more research data about the role of predictors of pharmacological and/or psychological treatment response can expand the current knowledge of these disorders and inform treatment decision making in clinical practice according to a precision medicine approach that offers to patients and their caregivers an optimal treatment strategy based upon their characteristics and healthcare needs (10–12). The predictors of pharmacological and/or psychological treatment response amongst patients with SAD and OCD can include patient-, family-, and treatment-level features which can predict or even moderate the outcome of patients or increase their readiness (i.e., willingness) to start a treatment.

The Research Topic entitled as “*Efficacy of Psychological and Psychiatric Treatments and Potential Predictors in Social Anxiety Disorder and Obsessive-Compulsive Disorder*” and edited by Rosa-Alcázar et al. aims to collect scientific papers focused on this clinical and research approach.

One of the most recently studied individual-level factor in SAD and OCD research regards the barriers against help-seeking intentions. For example, for SAD patients on the one hand, financial barriers, uncertainty over where to go for help, and fear of what others might think or say often prevent them from seeking treatment (13). OCD patients on the other hand can wait up to 10 years before seeking a professional help (14). In the online survey published in this Research Topic, carried out in South Africa by Hathorn et al. on 50 adults with OCD, more than one third of the sample endorsed wanting to handle the problem independently as a significant barrier, followed by treatment concerns (26%), affordability (22%), and shame (20%), and perceived treatment benefits were the only significant predictor of help-seeking intention. These data highlight the importance of public campaign and awareness programs aimed to reduce social stigma and increase the access of people with these conditions to evidence-based treatments.

Amongst the family-level variables which can negatively affect treatment drop-out and response in both the SAD and OCD population is represented by parents' or other informal caregivers' accommodation to the symptoms of the child, i.e., providing reassurance, assisting in avoidance, adjusting routines to patient's requests (15–17). In the Spanish study published by Rosa-Alcázar et al. in the current Research Topic, conducted on 56 children with OCD and their parents, mother's accommodation was associated with child's externalizing symptoms and initial severity of OCD, confirming the importance of addressing family accommodation in the therapeutic setting, particularly with the most severe patients. Although the authors highlighted a relationship with pre-treatment and posttreatment severity, mother's accommodation was not a mediating factor between initial severity and posttreatment after CBT and ERP and follow-up severity (Rosa-Alcázar et al.). This suggests the importance of assessing mother's accommodation in clinical practice; the fact that this variable was not a mediator perhaps suggests that standard CBT focused on symptoms but not including components specifically directed at family accommodation, might not be sufficient to affect this family mechanism; thus, this would call for the need of integrating a specific intervention aimed to target family accommodation in standard CBT.

With regards to treatment-level factors potentially influencing the efficacy of CBT in the field of SAD and OCD, new attention has been paid to the use of alternative treatment modalities. One of the most promising is mindfulness-based therapy (18, 19). In the randomized trial published within this Research Topic and conducted on 123 Chinese patients with OCD, Zhang et al. found that treatment outcomes were significantly better amongst those patients assigned to selective serotonin reuptake inhibitors or Mindfulness-Based Cognitive Therapy (MBCT) than amongst those assigned to psychoeducation about symptoms, whereas no significant differences emerged between the medications group and the MBCT group. However, there were no significant differences in treatment response amongst the three groups at 6 months follow-up. These promising data suggest that MBCT is a valid strategy that can be used as a monotherapy or be integrated in the standard CBT protocols and future research should examine its potential for the long-term stability of OCD symptom improvement.

In addition, another important treatment-level variable regards the efficacy of CBT for SAD and OCD during the pandemic, since this public health and socioeconomic change can be associated with an increase in OCD symptoms (20–22). In the study conducted in Italy by Zaccari et al. on 11 OCD patients who undertook cognitive therapy and ERP just before the lockdown between December 2019 and January 2020, an improvement in symptoms was noticed by 90% of the clinical sample and confirmed by 45% of the therapists, who claimed moderate progress in their patients. These preliminary data suggest that CBT could be a useful strategy for OCD also during the pandemic periods but studies with larger samples and controlled designs are required.

In summary, this Research Topic published in Frontiers in Psychiatry collects fours original papers on clinical child or adult individuals from different socio-cultural contexts around the world including Spain, South Africa, China, and Italy. The present papers focus on new research and clinical insights related to CBT for SAD and OCD and expand the knowledge about the patient-level barriers against help seeking of OCD patients such as the need for handling the problem independently as a significant barrier (Hathorn et al.), point out the role of family-level variables (i.e., mother's accommodation) with OCD children having the most severe symptoms (Rosa-Alcázar et al.), and treatment-level factors including the promising effectiveness of mindfulness-based therapies for OCD (Zhang et al.) and, the feasibility of CBT for OCD across the pandemic periods (Zaccari et al.).

In conclusion, the knowledge of the patient-, family- and treatment-level predictors of serotonergic and CBT response appear to be very important across all the phases of the therapeutic process of patients with SAD or OCD, from help seeking to follow-up, and in the context of the current pandemic, as well as the implementation of alternative strategies.

## Author Contributions

AP wrote the first draft of the editorial and revised the final version.

## Conflict of Interest

The author declares that the research was conducted in the absence of any commercial or financial relationships that could be construed as a potential conflict of interest.

## Publisher's Note

All claims expressed in this article are solely those of the authors and do not necessarily represent those of their affiliated organizations, or those of the publisher, the editors and the reviewers. Any product that may be evaluated in this article, or claim that may be made by its manufacturer, is not guaranteed or endorsed by the publisher.
